# Safety and Feasibility of Neuromuscular Electrical Stimulation in Patients with Extracorporeal Membrane Oxygenation

**DOI:** 10.3390/jcm13133723

**Published:** 2024-06-26

**Authors:** Christos Kourek, Vasiliki Raidou, Michael Antonopoulos, Maria Dimopoulou, Antigone Koliopoulou, Eleftherios Karatzanos, Theodoros Pitsolis, Konstantinos Ieromonachos, Serafim Nanas, Stamatis Adamopoulos, Themistocles Chamogeorgakis, Stavros Dimopoulos

**Affiliations:** 1 Clinical Ergospirometry, Exercise & Rehabilitation Laboratory, Evaggelismos Hospital, National and Kapodistrian University of Athens, 10676 Athens, Greece; chris.kourek.92@gmail.com (C.K.); vraidou@gmail.com (V.R.); magiad@hotmail.com (M.D.); lkaratzanos@gmail.com (E.K.); sernanas@gmail.com (S.N.); 2 Cardiac Surgery Intensive Care Unit, Onassis Cardiac Surgery Center, 17674 Kallithea, Greece; antonopoulos.m@hotmail.com (M.A.); theodorepitsolis@yahoo.com (T.P.); 3 Heart Failure, Transplant and Mechanical Circulatory Support Units, Onassis Cardiac Surgery Center, 17674 Athens, Greece; koliopoulou@ocsc.gr (A.K.); ieromonahos@ocsc.gr (K.I.); stamatis.adamo@gmail.com (S.A.); chamogeorgakis@ocsc.gr (T.C.)

**Keywords:** neuromuscular electrical stimulation (NMES), extracorporeal membrane oxygenation (ECMO), intensive care unit (ICU), safety, feasibility

## Abstract

**Background/Objectives**: The aim of this study was to investigate the feasibility and safety of neuromuscular electrical stimulation (NMES) in patients on extracorporeal membrane oxygenation (ECMO) and thoroughly assess any potential adverse events. **Methods**: We conducted a prospective observational study assessing safety and feasibility, including 16 ICU patients on ECMO support who were admitted to the cardiac surgery ICU from January 2022 to December 2023. The majority of patients were females (63%) on veno-arterial (VA)-ECMO (81%), while the main cause was cardiogenic shock (81%) compared to respiratory failure. Patients underwent a 45 min NMES session while on ECMO support that included a warm-up phase of 5 min, a main phase of 35 min, and a recovery phase of 5 min. NMES was implemented on vastus lateralis, vastus medialis, gastrocnemius, and peroneus longus muscles of both lower extremities. Two stimulators delivered biphasic, symmetric impulses of 75 Hz, with a 400 μsec pulse duration, 5 sec on (1.6 sec ramp up and 0.8 sec ramp down) and 21 sec off. The intensity levels aimed to cause visible contractions and be well tolerated. Primary outcomes of this study were feasibility and safety, evaluated by whether NMES sessions were successfully achieved, and by any adverse events and complications. Secondary outcomes included indices of rhabdomyolysis from biochemical blood tests 24 h after the application of NMES. **Results**: All patients successfully completed their NMES session, with no adverse events or complications. The majority of patients achieved type 4 and 5 qualities of muscle contraction. **Conclusions**: NMES is a safe and feasible exercise methodology for patients supported with ECMO.

## 1. Introduction

Neuromuscular electrical stimulation (NMES) is a modality of muscle exercise that applies electrical currents over muscles and nerves in a manner that produces smooth tetanic muscle contractions that simulate an exercise therapy session [1]. It has been used during the last two decades to improve muscle strength, prevent muscle atrophy, maintain the range of motion around a joint, and increase functional capacity through the activity of large muscle groups [2].

The implementation of NMES in stroke, heart failure, and chronic obstructive pulmonary disease rehabilitation has been shown as a promising alternative modality of exercise training [3,4,5]. There is also growing evidence that NMES may prevent intensive care acquired weakness (ICUAW), promoting early mobilization in critically ill patients with sepsis or those who require mechanical ventilation [6,7]. Prolonged mechanical ventilation duration, deep sedation, paralysis, long-term immobility, APACHE II, and lower albumin levels are the main risk factors for ICUAW in patients under ECMO support [8]. Major implications of ICUAW in ECMO patients include higher percentages of infection during ECMO support, liver and kidney dysfunction, prolonged hospital stay with greater financial burden, increased morbidity and mortality, and reduced quality of life [8]. In a recent systematic review, NMES was shown to be safe and feasible, and has beneficial effects on muscle strength and function in patients after cardiac surgery [9].

Extracorporeal membrane oxygenation (ECMO) is a short-term lifesaving mechanical organ support therapy for patients with severe respiratory failure, cardiogenic shock, or cardiac arrest refractory to conventional therapy. However, patients requiring ECMO support usually present ICUAW with significant muscle wasting and weakness with a high risk of post-intensive care syndrome that affect ECMO survivors’ quality of life [8,10]. 

Despite the potential beneficial effects of NMES implementation in ECMO patients, no relevant study has been reported so far to allow further investigation. 

Our study hypothesis was that the use of NMES in ECMO patients is safe and feasible. The aim of our study was to investigate the feasibility and safety of NMES for patients on ECMO and thoroughly assess any potential adverse events.

## 2. Materials and Methods

### 2.1. Study Design

This was an observational safety and feasibility prospective study assessing NMES in patients on ECMO support. This study was conducted in a cardiac surgery ICU in Athens, Greece, within a 2-year time period, from January 2022 to December 2023. This study was conducted in accordance with the ethical guidelines of the Declaration of Helsinki, and the Institutional Review Board (IBR) and the Ethics Committee of the Onassis Cardiac Surgery Center approved this study and associated data management (IRB number 664/12.12.2019, 12 December 2019, The effects of neuromuscular electrical stimulation in patients with extracorporeal membrane oxygenation support).

### 2.2. Patients

Patients were fully informed about the intervention, including potential benefits and risks, and a written informed consent form was obtained prior to the intervention from all patients or their next of kin. 

Inclusion criteria were (a) age ≥ 16 years and (b) hemodynamically and respiratory-stable patients post-ECMO implantation. 

ECMO indications included refractory cardiogenic shock post-cardiotomy, acute myocardial infarction, acute decompensated heart failure or myocarditis, primary graft dysfunction, and refractory respiratory insufficiency.

Cardiac surgery included coronary artery by-pass grafting (CABG); aortic and/or mitral and/or tricuspid valve replacement or plastic surgery; the Bentall operation; aortic aneurysm/dissection operation; or heart/lung transplantation.

All patients at enrollment fulfilled the following criteria prior to NMES sessions: a mean arterial blood pressure greater than 65 mm Hg; use of vasopressors at <50% of the maximum dose; a heart rate between 50 and 140 bpm, without arrhythmias with hemodynamic consequences or myocardial ischemia; a temperature between 36 and 38 °C; and a stable peripheral oxygen saturation (SpO_2_) baseline value (>88%).

Exclusion criteria included patients who had active tissue infection in areas where electrodes would be placed; pregnant women; patients with a body mass index greater than 35 kg/m^2^; other contraindications of NMES, including fractures, burns, skin lesions, thromboembolic disease, deep vein thrombosis, and lower limb amputations; and patients on left ventricular assist devices (LVADs). 

All ECMO patients received ECMO configurations according to our institute’s protocol. Peripheral veno-arterial ECMO was implanted through femoral vessels cannulation (21/23Fr venous, 17/19Fr arterial cannulas, Medtronic) with an antegrade distal perfusion catheter (7F, sheath, Arrow) to avoid lower limb ischemia and peripheral veno-venous ECMO through femoral (drainage cannula, 21/25F, Medtronic) and jugular vessel cannulation (return cannula, 17/19F, Medtronic).

### 2.3. NMES Protocol

**Figure 1:**
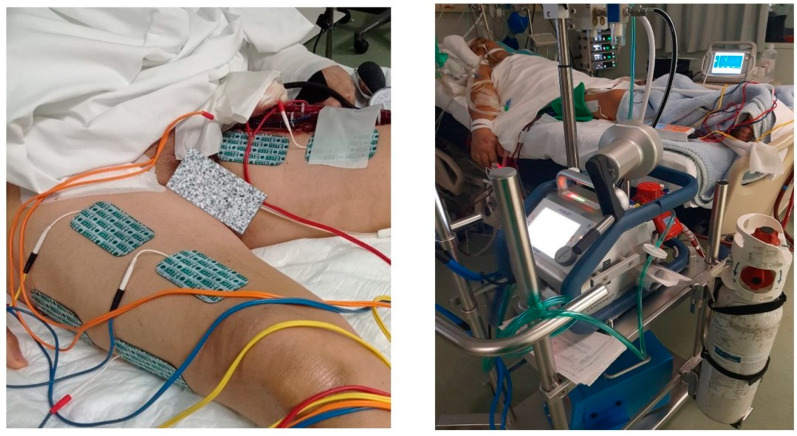
NMES session of a patient under VA-ECMO support in the cardiac surgery ICU.

NMES was implemented on vastus lateralis, vastus medialis, gastrocnemius, and peroneus longus muscles of both lower extremities. Specifically, 16 rectangular electrodes (90 × 50 mm) in total were placed on the motor points of vastus lateralis, vastus medialis, and peroneus longus of both lower extremities after appropriate skin preparation, including shaving and skin cleaning. Each session had a duration of 45 min and included a warm-up phase of 5 min, a main phase of 35 min, and a recovery phase of 5 min. We used two stimulators (4-channel T-One Medi Sport Electric stimulator, I-TECH Medical Division, Scorzè, Italy) that delivered biphasic, symmetric impulses of 75 Hz, with a 400 μsec pulse duration, 5 sec on (1.6 sec ramp up and 0.8 sec ramp down) and 21 sec off. The intensity levels aimed to cause visible contractions and be tolerated by patients. In case of doubt, contractions were confirmed by palpation (Figure 1). The angle of the patients’ knee joints was approximately 40° (0° corresponds to full knee extension).

The quality of muscle contraction was rated using a 5-point scoring system, as follows: (type 1) no palpable or visible contraction, (type 2) just palpable but no visible contraction, (type 3) just palpable and visible contraction, (type 4) palpable and visible contraction (partial muscle bulk), and (type 5) palpable and visible contraction (full muscle bulk) [11,12]. The target quality of contraction was type 3 during warm-up and recovery phases and type 4 or 5 during the main phase.

### 2.4. Outcomes

Primary outcomes of this study were feasibility and safety, evaluated by whether the NMES sessions were successfully achieved, and by any adverse events and complications, such as severe arrhythmias, hypotension or dizziness, decrease in saturation, signs of pain during electrical stimulation, muscle damage, cardiogenic shock, or decannulation during NMES implementation. Adverse events were evaluated during NMES sessions by continuous monitoring of the vital signs of patients and patients’ verbal expressions of pain or discomfort if non-sedated, otherwise by facial expressions if mildly sedated. Adverse events were also evaluated after NMES sessions by continuous monitoring of vital signs for several hours, as well as by indices of the arterial blood gas and indices of rhabdomyolysis in biochemical blood tests 24 h after NMES. Another significant and frequent adverse effect of transcutaneous electric stimulation that we assessed was electric burn, and, in fact, the inability of sedated patients to express pain from burn may be a relative contraindication of NMES. In order to prevent NMES burns, we performed daily care of NMES electrodes and replaced any failed electrodes, we checked irritation and redness of the skin around the electrodes during and after NMES, and we applied low pulse duration. The quality of muscle contraction was also rated at four time points during each NMES session; once at the warm-up, twice at the main phase, and once at the recovery phase.

Secondary outcomes were pH and lactate from arterial blood gas tests, as well as indices of muscle damage from biochemical blood tests, including creatine phosphokinase (CPK), aspartate aminotransferase (AST), alanine aminotransferase (ALT), and lactate dehydrogenase (LDH), which were measured before and 24 h after the application of NMES.

### 2.5. Statistical Analysis

Normality of distribution was checked with the Shapiro–Wilk test. Variables are expressed as frequency and percentage, mean ± standard deviation (SD), or median (25th–75th percentiles). A paired two-sample Student’s *t*-test analyzed differences in dependent parameters with normal distribution, while the Wilcoxon signed-rank test analyzed differences in nonparametric data. Differences between time points were assessed with repeated measures factorial analysis of variance (ANOVA). All tests were two-tailed and the level of statistical significance was set at 0.05. Statistical analyses were performed with IBM SPSS 25 Statistics software (Armonk, NY, USA).

## 3. Results

Demographics and clinical data regarding ECMO patients are demonstrated in detail in Table 1. Initially, 16 patients met the inclusion criteria to enroll in this study. However, two patients were finally excluded: the first patient presented significant edema of the lower limbs and no contraction could be observed during NMES, while the second patient experienced a brief episode of intense anxiety and we avoided performing NMES and deteriorating the situation. Our final sample size in the analysis of parameters of interest consisted of 14 ICU patients on ECMO support. 

No adverse events or complications due to NMES were observed. No electric burns due to transcutaneous electric stimulation were presented in patients on ECMO. Median intensities of the electrical stimulation for both limbs according to NMES phases are presented in Table 1. With regards to the quality of muscle contraction, the majority of patients achieved type 4 and 5 contractions (Table 1).

All patients successfully completed their 45 min NMES session, without presenting hemodynamic deterioration or any event such as hypotension, severe arrhythmia, desaturation, or discomfort. Hemodynamic parameters did not change before, during (at 5 min, 35 min, and 40 min), or after NMES sessions (Table 2). Arterial blood gas indices, including pH and lactate, remained unchanged at two time points (before and after NMES, Table 2). Finally, biochemical blood tests did not show any difference in CPK, AST, or LDH 24 h after the NMES session, indicating that there were no signs of rhabdomyolysis (muscle damage) due to the intervention. ALT significantly decreased 24 h after the intervention (Table 2).

## 4. Discussion

Findings of our study show that NMES implementation is safe and feasible in patients on ECMO support. To the best of our knowledge, our study is the first to demonstrate NMES safety and feasibility in ECMO patients, assessing adverse events and complications and also respiratory, hemodynamic, and biochemical parameters, and arterial blood gas indices before and after NMES sessions.

There has been only a single crossover feasibility case study in the literature, in which the authors investigated the effects of NMES applied to the quadriceps muscle on the pedal perfusion of patients receiving veno-venous ECMO (VV-ECMO) via bifemoral cannulation [13]. 

In accordance to our findings, NMES applications seemed to be well tolerated, with no alteration to ECMO flows, sedation requirements, or observed cardiovascular parameters, indicating that NMES may be a viable exercise modality in bifemorally cannulated VV-ECMO patients who cannot perform early mobilization exercises or other modalities of exercise. However, the sample size was quite small, including only three patients, and, as a result, no safe conclusions could be extracted from that study [13]. In the present study, we have demonstrated further that NMES can be safely applied in both VV-ECMO (femoro-jugular configuration) and VA-ECMO (femoro-femoral configuration) patients.

ICUAW is a common complication of critically ill patients on ECMO and patients on ECMO as a bridge to lung/heart transplantation or as a bridge to recovery in the ICU, leading to persistent functional disability and decreased quality of life after hospital discharge [14]. The major risk factors for ICUAW are immobilization, multiple organ failure, systemic inflammatory response syndrome, gram-negative septicemia [15], hyperglycemia [16,17], and medications such as aminoglycosides and corticosteroids. There are various proposed modalities of early rehabilitation, especially for patients on ECMO, that have the potential to prevent skeletal–muscular weakness and wasting in critically ill patients [18,19]. These interventions include passive mobilization via stretching, splinting and passive movements; continuous passive motion; functional electrical stimulation of lower limb muscles and passive cycling for unconscious patients of higher severity or intubated patients; active range-of-motion and resistance exercises, such as leg presses, squats from a sitting position, and active cycling; mobilization in a standing position and walking; and respiratory rehabilitation and breathing exercises for awake patients with a satisfactory level of consciousness who can follow orders or provide feedback [19]. 

NMES is an exercise modality that can be applied even in sedated critically ill patients [9]. A NMES prescription includes the NMES frequency in Hz, which is the number of pulses in one second (20–50 pulses per second); the pulse duration in microseconds (for small muscles, approximately 150–200, and for large muscles, 200–300); the ramp time, which is at least 2 sec; the exercise/recovery time ratio, which should be set to allow enough time for muscle recovery from exercise; the treatment session duration, which should be at least 20 to 30 min; and the frequency of sessions, which should occur at least three times per week when applied systemically for a patient [1,20]. NMES normally involves frequencies between 20 and 50 Hz and pulse widths between 200 and 400 μsec [21], and should recruit motor axons preferentially to sensory axons [22]. A higher exercise/recovery ratio (1:5) provides more rest time between muscle contractions and causes less muscle fatigue [1]. In our study, we used biphasic, symmetric impulses of 75 Hz, with a 400 μsec pulse duration, 5 sec on (1.6 sec ramp up and 0.8 sec ramp down) and 21 sec off, based on previous studies [23,24]. Although intensity levels were higher than suggested, we aimed to cause visible contractions that were tolerated by patients. Moreover, our higher exercise/recovery time ratio may have prevented muscle fatigue by providing more rest time between muscle contractions.

Motor impairment and activity limitation have been shown to reduce after the implementation of NMES, but without a full understanding of its potential mechanisms. One hypothesis is that therapeutic effects of NMES are probably due to a combination of peripheral cardiovascular and musculoskeletal effects. Peripheral effects of NMES include an increase in contractile force and fatigue resistance [25,26], an increase in muscle mass [27], a reduction in edema [28], the conversion of fast-twitch fast-fatiguing glycolytic type II muscle fibers to slow-twitch fatigue-resistant oxidative type I muscle fibers [26], and enhanced hyperemic arterial response and endothelium-dependent cutaneous vasodilation [29]. Another beneficial effect of NMES may be the fact that it affects the central nervous system and promotes motor relearning by uniquely providing an artificial method of ensuring synchronized presynaptic and postsynaptic activity (Hebbian plasticity), especially if the electrical stimulation is paired with simultaneous voluntary effort that activates residual upper motor neurons [30]. This mechanism of NMES could be quite useful for patients’ rehabilitation after stroke. Severe systematic inflammation, along with electrolyte changes, as well as intense edema, may seriously affect conductivity, and thus electrical current diffusion [31], and could reduce any systemic effect of NMES. Indeed, in our study we excluded one patient due to the severe edema he presented in the lower limbs. However, a recent systematic review supports the use of NMES for ameliorating edema, indicating that it could be effective even in the treatment of both upper and lower limb edema [32]. This could be a future direction for randomized controlled trials, in order to investigate the treatment effect of NMES in patients with upper and lower limb edema.

Similar studies assessing NMES in critically ill patients were previously performed by our research group. Specifically, in 2013, Angelopoulos E. et al. [23] compared the effects on muscle microcirculation of a single NMES session using medium frequency currents of 45 Hz and high frequency currents of 75 Hz in ICU patients with systemic inflammatory response syndrome (SIRS) or sepsis. That study showed that a single NMES session affected local and systemic skeletal muscle microcirculation by improving endothelial reactivity and vascular reserve at both frequencies, with equally effective results between medium and high frequency currents. Interestingly, NMES has been also shown to promote endothelial progenitor cells (EPCs) after NMES sessions in critically ill patients, indicating its potential beneficial effects on endothelial injury [24].

This was a safety and feasibility study with some limitations. The sample size was small, including 16 patients on ECMO in the ICU, and thus, safety and feasibility conclusions cannot be easily generalizable to the whole ECMO population. However, it is remarkable that we did not observe any adverse event, and NMES was well tolerated by all patients. A randomized controlled trial with more patients on ECMO support will provide more evidence regarding NMES’ potentially beneficial effects. In our study, we performed a single NMES session, and thus, we did not investigate the chronic effect of NMES after regular implementation in these patients; however, this study was not designed to test NMES efficacy. 

Future perspectives regarding NMES in patients on ECMO include randomized trials evaluating its impact on the function and muscle strength of the upper and lower extremities of these patients. Moreover, the appropriate frequency and intensity of NMES needs to be further evaluated and individualized for each patient in order to provide the best possible effect and prevent ICUAW. Another significant future perspective is the investigation of the effectiveness of cardiopulmonary rehabilitation on the rate of ready for ECMO weaning in ECMO-supported patients [33]. The main problem for critically ill ECMO-supported patients is the fact that the majority fail to wean from ECMO therapy. Annual ECMO mortality has been reported to be approximately 40–70% [34], which is extremely high. Moreover, delayed ECMO weaning demonstrates the worst functional prognosis and impaired health-related quality of life [33]. Early ECMO weaning would improve outcomes, reduce cost, and optimize functional prognosis [35]. Thus, the investigation whether cardiopulmonary rehabilitation may subsequently contribute to earlier weaning from ECMO seems to be significant for better clinical outcomes.

## 5. Conclusions

In conclusion, findings of our study have shown that NMES is a safe and feasible exercise methodology for patients supported with ECMO. Further studies are required to assess NMES’ efficacy as a valid preventive and therapeutic tool for ICUAW, promoting early mobilization of these patients.

## Figures and Tables

**Table 1:** Demographic characteristics for patients under ECMO support who underwent a single NMES session.

Demographic Characteristics	
Number of patients (N)	16
Gender (Females)	10 (63%)
Age (years) ^a^	46.6 ± 13.7
BMI (kg/m^2^) ^a^	25.8 ± 3.1
**Cause of ECMO**	
Cardiogenic shock	13 (81%)
Respiratory failure	3 (19%)
**ECMO parameters**	
FiO_2_ (%) ^a^	71.6 ± 19.1
ECMO pump flow (L/min) ^a^	2.62 ± 1.03
RPM (revolutions per min) ^a^	2478.0 ± 611.5
**Type of ECMO**	
VA-ECMO (femo-femoral)	13 (81%)
VV-ECMO (femo-jugular)	3 (19%)
**Duration of ECMO (days)** ^b^	8 (5–28.75)
**Ventilation during NMES**	13 (81%)
**Sedation during NMES**	9 (56%)
**Type of muscle contraction** ^b^	
Warm-up	4 (3–4)
Main phase	5 (4–5)
Recovery phase	4 (2–4)
**Amplitude of NMES** ^b^	
Warm-up (mA)	30 (20–55)
Main phase (mA)	53 (30–86)
Recovery phase (mA)	43 (36–74)

ECMO, extracorporeal membrane oxygenation; BMI, body mass index; NMES, neuromuscular electrical stimulation; FiO_2_, fraction of inspired oxygen of ECMO circuit; RPM, pump speed of the ECMO circuit; VV-ECMO, veno-venous ECMO; VA-ECMO, veno-arterial ECMO; SD, standard deviation. ^a^ Values are expressed as mean ± SD. ^b^ Values are expressed as median (25th–75th percentiles).

**Table 2:** Hemodynamic, respiratory, and biochemical parameters and arterial blood gas indices at different time points, before, during, and after NMES sessions.

Hemodynamic Parameters ^a^		During NMES			
	Before NMES	5 min	35 min	40 min	*p* Value
Systolic BP (mmHg)	114.1 ± 26.7	114.8 ± 22.3	114.4 ± 25.2	112.2 ± 21.1	0.484
Diastolic BP (mmHg)	68.4 ± 15.4	67.6 ± 17.5	66.8 ± 15.9	65.9 ± 16.1	0.181
Mean BP (mmHg)	82.1 ± 18.2	82.6 ± 15.3	82.7 ± 16.4	82.0 ± 16.0	0.964
HR (beats/min)	102.4 ± 19.7	101.1 ± 20.1	101.4 ± 20.5	100.2 ± 19.5	0.475
SpO_2_ (%)	95.1 ± 10.4	96.7 ± 3.8	97.2 ± 3.4	97.2 ± 3.2	0.442
RR (breaths/min)	17.3 ± 6.2	18.3 ± 5.5	18.9 ± 6.7	17.9 ± 6.2	0.457
**ABG indices** ^a^	**Before NMES**		**After NMES**		***p* value**
Arterial blood pH	7.43 ± 0.07		7.45 ± 0.06		0.468
Lactate (mmol/L)	1.68 ± 0.78		1.44 ± 0.60		0.242
**Biochemical parameters** ^b^	**Before NMES**		**24 h after NMES**		***p* value**
CPK (IU/L)	327.5 (113.0–1234.5)		257.5 (129.5–637.5)		0.657
AST-SGOT (IU/L)	70.0 (32.0–118.8)		57.5 (33.0–110.3)		0.470
ALT-SGPT (IU/L)	50.0 (25.3–280.5)		43.0 (23.8–136.3)		**0.006**
LDH (IU/L)	488.0 (288.0–776.5)		556.5 (321.5–756.8)		0.972

ECMO, extracorporeal membrane oxygenation; BMI, body mass index; NMES, neuromuscular electrical stimulation; BP, blood pressure; SpO_2_, oxygen saturation; HR, heart rate; RR, respiratory rate; ABG, arterial blood gas; CPK, creatine phosphokinase; AST, aspartate aminotransferase; SGOT, serum glutamic-oxaloacetic transaminase; ALT, alanine aminotransferase; SGPT, serum glutamic-pyruvic transaminase; LDH, lactate dehydrogenase; SD, standard deviation. ^a^ Values are expressed as mean ± SD. ^b^ Values are expressed as median (25th–75th percentiles).

## Data Availability

Data are available upon request.
